# Correction: Regression approaches for modeling genotype‑environment interaction and making predictions into unseen environments

**DOI:** 10.1007/s00122-026-05188-8

**Published:** 2026-04-06

**Authors:** Maksym Hrachov, Hans-Peter Piepho, Niaz Md. Farhat Rahman, Waqas Ahmed Malik

**Affiliations:** 1 Biostatistics Unit, Institute of Crop Science, University of Hohenheim, 70593 Stuttgart, Germany; 2 Bangladesh Rice Research Institute (BRRI), Gazipur, Bangladesh

**Correction to: Theoretical and Applied Genetics (2026) 139:32** 10.1007/s00122-025-05103-7

In this published article, the math symbols and special characters were wrongly substituted in several places due to typesetting mistake.

The original article has been corrected.

