# Supplementary material for: Regression approaches for modeling genotype-environment interaction and making predictions into unseen environments
Source: Theor Appl Genet. 2026 Jan 12;139(1):32. doi: 10.1007/s00122-025-05103-7 (PMC12791071; doi:10.1007/s00122-025-05103-7)
Supplement: Supplementary file 1 — Supplementary file1 (DOCX 253 KB) [file 122_2025_5103_MOESM1_ESM.docx]

**Regression approaches for modelling genotype-environment interaction and making predictions into unseen environments**

Maksym Hrachov^1^, Hans-Peter Piepho^1^, Niaz Md. Farhat Rahman^2^, Waqas Ahmed Malik^1^

1 Biostatistics Unit, Institute of Crop Science, University of Hohenheim, Stuttgart, Germany

2 Bangladesh Rice Research Institute (BRRI), Gazipur, Bangladesh

*Theoretical and Applied Genetics (TAG)*

**Correspondence**

Hans-Peter Piepho, Biostatistics Unit, Institute of Crop Science, University of Hohenheim, 70593 Stuttgart, Germany.

Email: piepho@uni-hohenheim.de

**SUPPLEMENTARY INFORMATION**

**Table S1.** Model fit (winter rice) featuring the total number of parameters in the model, including those involved in synthetic covariates, full log-likelihood, and Akaike Information Criterion (AIC). Displayed values are rounded. Baseline – model without genotype-covariate interactions, Kernel – model with an environmental kernel matrix, RRR1 and RRR2 – reduced rank regression of rank one and two with observed covariates, FW1-US and FW2-US – random factorial regression with one and two synthetic covariates respectively, RFR – random factorial regression with observed covariates.

| **With the main EC effect** | | | | **Without the main EC effect** | | | |
| --- | --- | --- | --- | --- | --- | --- | --- |
| **Model** | **Parameters** | **LogLik** | **AIC** | **Model** | **Parameters** | **LogLik** | **AIC** |
| Baseline | 16 | -542 | 1117 | Baseline | 8 | -553 | 1122 |
| Kernel | 17 | -542 | 1119 | Kernel | 9 | -553 | 1124 |
| RRR1 | 24 | -525 | 1098 | RRR1 | 16 | -536 | 1104 |
| RRR2 | 32 | -505 | 1074 | RRR2 | 24 | -516 | 1079 |
| RFR | 60 | -496 | 1113 | RFR | 52 | -507 | 1118 |
| FW1-US | 19 | -519 | 1077 | FW1-US | 18 | -524 | 1084 |
| FW2-US | 30 | -507 | 1074 | FW2-US | 28 | -513 | 1082 |

**Table S2.** Variance components of models (summer rice). Baseline – model without genotype-covariate interactions, Kernel – model with an environmental kernel matrix, RRR1 and RRR2 – reduced rank regression of rank one and two with observed covariates, FW1-US and FW2-US – random factorial regression with one and two synthetic covariates respectively, RFR – random factorial regression with observed covariates, L – location, Y – year, α – genotype.

| **Models with the main EC effect** | | | | | | | |
| --- | --- | --- | --- | --- | --- | --- | --- |
| **Component** | **Baseline** | **Kernel** | **RRR1** | **RRR2** | **RFR** | **FW1-US** | **FW2-US** |
| *L* | 0.0568 | 0.0554 | 0.0557 | 0.0536 | 0.0537 | 0.1016 | 0.0535 |
| *Y* | 0.0262 | 0.0270 | 0.0257 | 0.0287 | 0.0287 | 0.0237 | 0.0370 |
| *α* | 0.2422 | 0.2438 | 0.2309 | 0.2321 | 0.2291 | 0.2339 | 0.2345 |
| *LY* | 0.4434 | 0.4442 | 0.4446 | 0.4466 | 0.4462 | 0.4476 | 0.4429 |
| *αL* | 0.0307 | 0.0225 | 0.0268 | 0.0223 | 0.0186 | 0.0249 | 0.0218 |
| *αY* | 0.0142 | 0.0108 | 0.0120 | 0.0071 | 0.0060 | 0.0097 | 0.0096 |
| *αLY* | 0.2695 | 0.2693 | 0.2706 | 0.2678 | 0.2668 | 0.2667 | 0.2666 |
| **Models without the main EC effect** | | | | | | | |
| **Component** | **Baseline** | **Kernel** | **RRR1** | **RRR2** | **RFR** | **FW1-US** | **FW2-US** |
| *L* | 0.1269 | 0.1275 | 0.1295 | 0.1301 | 0.1308 | 0.1280 | 0.1299 |
| *Y* | 0.0250 | 0.0263 | 0.0274 | 0.0321 | 0.0320 | 0.0300 | 0.0297 |
| *α* | 0.2430 | 0.2450 | 0.2328 | 0.2344 | 0.2316 | 0.2352 | 0.2359 |
| *LY* | 0.4441 | 0.4448 | 0.4459 | 0.4475 | 0.4473 | 0.4459 | 0.4472 |
| *αL* | 0.0307 | 0.0226 | 0.0269 | 0.0221 | 0.0186 | 0.0248 | 0.0218 |
| *αY* | 0.0142 | 0.0108 | 0.0119 | 0.0071 | 0.0060 | 0.0097 | 0.0095 |
| *αLY* | 0.2695 | 0.2693 | 0.2706 | 0.2678 | 0.2669 | 0.2668 | 0.2667 |

**Table S3.** Variance components of models (winter rice). Baseline – model without genotype-covariate interactions, Kernel – model with an environmental kernel matrix, RRR1 and RRR2 – reduced rank regression of rank one and two with observed covariates, FW1-US and FW2-US – random factorial regression with one and two synthetic covariates respectively, RFR – random factorial regression with observed covariates, L – location, Y – year, α – genotype.

| **Models with the main EC effect** | | | | | | | |
| --- | --- | --- | --- | --- | --- | --- | --- |
| **Component** | **Baseline** | **Kernel** | **RRR1** | **RRR2** | **FW1-US** | **FW2-US** | **RFR** |
| *L* | 0.1530 | 0.1530 | 0.1531 | 0.1537 | 0.1529 | 0.1987 | 0.1776 |
| *Y* | 0.0381 | 0.0381 | 0.0383 | 0.0391 | 0.0388 | 0.0361 | 0.0301 |
| *α* | 0.1331 | 0.1331 | 0.1034 | 0.1119 | 0.1152 | 0.1115 | 0.1104 |
| *LY* | 0.4932 | 0.4932 | 0.4928 | 0.4930 | 0.4934 | 0.5030 | 0.5077 |
| *αL* | 0.0310 | 0.0310 | 0.0304 | 0.0308 | 0.0274 | 0.0321 | 0.0304 |
| *αY* | 0.0148 | 0.0148 | 0.0134 | 0.0058 | 0.0041 | 0.0066 | 0.0051 |
| *αLY* | 0.2593 | 0.2593 | 0.2583 | 0.2584 | 0.2569 | 0.2591 | 0.2573 |
| **Models without the main EC effect** | | | | | | | |
| *L* | 0.2126 | 0.2126 | 0.2160 | 0.2156 | 0.2135 | 0.2129 | 0.2155 |
| *Y* | 0.0435 | 0.0435 | 0.0468 | 0.0433 | 0.0425 | 0.0443 | 0.0445 |
| *α* | 0.1338 | 0.1338 | 0.1051 | 0.1154 | 0.1185 | 0.1146 | 0.1135 |
| *LY* | 0.5198 | 0.5198 | 0.5210 | 0.5184 | 0.5187 | 0.5202 | 0.5208 |
| *αL* | 0.0310 | 0.0309 | 0.0303 | 0.0309 | 0.0274 | 0.0322 | 0.0305 |
| *αY* | 0.0148 | 0.0147 | 0.0134 | 0.0057 | 0.0040 | 0.0064 | 0.0050 |
| *αLY* | 0.2593 | 0.2593 | 0.2583 | 0.2584 | 0.2569 | 0.2591 | 0.2573 |

**Table S4.** Variance components present change relative to the baseline (winter rice). Baseline – model without genotype-covariate interactions, Kernel – model with an environmental kernel matrix, RRR1 and RRR2 – reduced rank regression of rank one and two with observed covariates, FW1-US and FW2-US – random factorial regression with one and two synthetic covariates respectively, RFR – random factorial regression with observed covariates, L – location, Y – year, α – genotype.

| **Models with the main EC effect** | | | | | | | |
| --- | --- | --- | --- | --- | --- | --- | --- |
| **Component** | **Baseline**^a^ | **Kernel** | **RRR1** | **RRR2** | **RFR** | **FW1-US** | **FW2-US** |
| *L* | 0.1530 | 0.0 | 0.1 | 0.5 | -0.1 | 29.9 | 16.1 |
| *Y* | 0.0381 | 0.0 | 0.5 | 2.7 | 1.9 | -5.2 | -20.9 |
| *α* | 0.1331 | 0.0 | -22.3 | -15.9 | -13.4 | -16.3 | -17.0 |
| *LY* | 0.4932 | 0.0 | -0.1 | 0.0 | 0.0 | 2.0 | 2.9 |
| *αL* | 0.0310 | -0.1 | -1.9 | -0.6 | -11.7 | 3.5 | -1.9 |
| *αY* | 0.0148 | -0.1 | -9.3 | -60.5 | -72.0 | -55.5 | -65.5 |
| *αLY* | 0.2593 | 0.0 | -0.4 | -0.3 | -0.9 | -0.1 | -0.8 |
| **Models without the main EC effect** | | | | | | | |
| **Component** | **Baseline**^a^ | **Kernel** | **RRR1** | **RRR2** | **RFR** | **FW1-US** | **FW2-US** |
| *L* | 0.2126 | 0.0 | 1.6 | 1.4 | 0.4 | 0.1 | 1.4 |
| *Y* | 0.0435 | 0.0 | 7.7 | -0.4 | -2.2 | 1.9 | 2.3 |
| *α* | 0.1338 | 0.0 | -21.5 | -13.8 | -11.5 | -14.4 | -15.2 |
| *LY* | 0.5198 | 0.0 | 0.2 | -0.3 | -0.2 | 0.1 | 0.2 |
| *αL* | 0.0310 | -0.1 | -2.1 | -0.2 | -11.4 | 3.9 | -1.5 |
| *αY* | 0.0148 | -0.1 | -9.3 | -61.6 | -72.8 | -56.3 | -66.4 |
| *αLY* | 0.2593 | 0.0 | -0.4 | -0.5 | -1.2 | -0.2 | -0.8 |

^a^ Baseline represents the actual values from the Baseline model, the rest are in percent relative to it.

**Table S5.** Leave-one-environment-out (LOEO) and leave-one-year-and-location-out (LYLO) cross-validation means (winter rice). PCC – Pearson's correlation coefficient, MSEPD – mean squared error of prediction difference, MSPE – mean squared prediction error, Baseline – model without genotype-covariate interactions, Kernel – model with an environmental kernel matrix, RRR1 and RRR2 – reduced rank regression of rank one and two with observed covariates, FW1-US and FW2-US – random factorial regression with one and two synthetic covariates, RFR – random factorial regression with observed covariates.

| **Type** | **Model** | **Mean PCC** | | **Mean MSEPD_­_** | | **Mean MSPE** | |
| --- | --- | --- | --- | --- | --- | --- | --- |
|  |  | **LOEO** | **LYLO** | **LOEO** | **LYLO** | **LOEO** | **LYLO** |
| With the main EC effect | Baseline | 0.466 | 0.400 | 0.780 | 0.838 | 0.960 | 1.22 |
|  | Kernel | 0.466 | 0.400 | 0.780 | 0.838 | 0.960 | 1.21 |
|  | RRR1 | 0.468 | 0.393 | 0.780 | 0.846 | 0.960 | 1.22 |
|  | RRR2 | 0.465 | 0.389 | 0.779 | 0.850 | 0.962 | 1.22 |
|  | RFR | 0.462 | 0.389 | 0.782 | 0.849 | 0.964 | 1.22 |
|  | FW1-US | 0.468 | 0.391 | 0.777 | 0.847 | 0.957 | 1.22 |
|  | FW2-US | 0.465 | 0.389 | 0.780 | 0.85 | 0.962 | 1.23 |
| Without the main EC effect | Baseline | 0.466 | 0.400 | 0.780 | 0.838 | 0.969 | 1.22 |
|  | Kernel | 0.466 | 0.400 | 0.780 | 0.838 | 0.969 | 1.22 |
|  | RRR1 | 0.468 | 0.393 | 0.780 | 0.846 | 0.971 | 1.22 |
|  | RRR2 | 0.465 | 0.389 | 0.779 | 0.850 | 0.968 | 1.22 |
|  | RFR | 0.462 | 0.389 | 0.782 | 0.849 | 0.970 | 1.22 |
|  | FW1-US | 0.467 | 0.391 | 0.777 | 0.847 | 0.966 | 1.22 |
|  | FW2-US | 0.465 | 0.388 | 0.780 | 0.850 | 0.970 | 1.22 |

**Table S6.** Leave-one-environment-out (LOEO) and leave-one-year-and-location-out (LYLO) cross-validation medians (winter rice). PCC – Pearson's correlation coefficient, MSEPD – mean squared error of prediction difference, MSPE – mean squared prediction error, Baseline – model without genotype-covariate interactions, Kernel – model with an environmental kernel matrix, RRR1 and RRR2 – reduced rank regression of rank one and two with observed covariates, FW1-US and FW2-US – random factorial regression with one and two synthetic covariates, RFR – random factorial regression with observed covariates.

| **Type** | **Model** | **Median PCC** | | **Median MSEPD_­_** | | **Median MSPE** | |
| --- | --- | --- | --- | --- | --- | --- | --- |
|  |  | **LOEO** | **LYLO** | **LOEO** | **LYLO** | **LOEO** | **LYLO** |
| With the main EC effect | Baseline | 0.545 | 0.438 | 0.661 | 0.711 | 0.620 | 0.744 |
|  | Kernel | 0.545 | 0.435 | 0.661 | 0.712 | 0.620 | 0.797 |
|  | RRR1 | 0.544 | 0.430 | 0.658 | 0.701 | 0.622 | 0.816 |
|  | RRR2 | 0.547 | 0.433 | 0.664 | 0.723 | 0.653 | 0.810 |
|  | RFR | 0.553 | 0.427 | 0.678 | 0.722 | 0.634 | 0.804 |
|  | FW1-US | 0.546 | 0.442 | 0.646 | 0.722 | 0.631 | 0.863 |
|  | FW2-US | 0.540 | 0.430 | 0.692 | 0.731 | 0.643 | 0.851 |
| Without the main EC effect | Baseline | 0.546 | 0.437 | 0.661 | 0.711 | 0.658 | 0.824 |
|  | Kernel | 0.546 | 0.435 | 0.661 | 0.712 | 0.658 | 0.824 |
|  | RRR1 | 0.544 | 0.431 | 0.657 | 0.702 | 0.664 | 0.851 |
|  | RRR2 | 0.547 | 0.432 | 0.665 | 0.725 | 0.633 | 0.851 |
|  | RFR | 0.553 | 0.427 | 0.677 | 0.723 | 0.630 | 0.839 |
|  | FW1-US | 0.544 | 0.443 | 0.647 | 0.723 | 0.642 | 0.846 |
|  | FW2-US | 0.539 | 0.431 | 0.693 | 0.734 | 0.634 | 0.846 |

**Table S7.** Leave-one-environment-out (LOEO) and leave-one-year-and-location-out (LYLO) cross-validation medians (summer rice). PCC – Pearson's correlation coefficient, MSEPD – mean squared error of prediction difference, MSPE – mean squared prediction error, Baseline – model without genotype-covariate interactions, Kernel – model with an environmental kernel matrix, RRR1 and RRR2 – reduced rank regression of rank one and two with observed covariates, FW1-US and FW2-US – random factorial regression with one and two synthetic covariates, RFR – random factorial regression with observed covariates.

| **Type** | **Model** | **Median PCC** | | **Median MSEPD_­_** | | **Median MSPE** | |
| --- | --- | --- | --- | --- | --- | --- | --- |
|  |  | **LOEO** | **LYLO** | **LOEO** | **LYLO** | **LOEO** | **LYLO** |
| With the main EC effect | Baseline | 0.697 | 0.665 | 0.645 | 0.699 | 0.576 | 0.656 |
|  | Kernel | 0.695 | 0.671 | 0.617 | 0.697 | 0.570 | 0.645 |
|  | RRR1 | 0.695 | 0.668 | 0.665 | 0.709 | 0.569 | 0.660 |
|  | RRR2 | 0.691 | 0.671 | 0.654 | 0.720 | 0.581 | 0.665 |
|  | RFR | 0.693 | 0.661 | 0.652 | 0.720 | 0.575 | 0.665 |
|  | FW1-US | 0.694 | 0.669 | 0.648 | 0.717 | 0.556 | 0.642 |
|  | FW2-US | 0.694 | 0.669 | 0.665 | 0.716 | 0.605 | 0.625 |
| Without the main EC effect | Baseline | 0.697 | 0.665 | 0.645 | 0.699 | 0.584 | 0.644 |
|  | Kernel | 0.695 | 0.670 | 0.618 | 0.697 | 0.582 | 0.652 |
|  | RRR1 | 0.695 | 0.668 | 0.665 | 0.711 | 0.582 | 0.650 |
|  | RRR2 | 0.691 | 0.670 | 0.654 | 0.720 | 0.567 | 0.672 |
|  | RFR | 0.693 | 0.662 | 0.653 | 0.720 | 0.561 | 0.678 |
|  | FW1-US | 0.694 | 0.668 | 0.648 | 0.716 | 0.571 | 0.671 |
|  | FW2-US | 0.694 | 0.668 | 0.665 | 0.716 | 0.572 | 0.672 |

**Table S8.** Mean variance of prediction (MVP) and mean squared prediction error (MSPE) from leave-one-year-and-location-out cross-validation (winter rice). Baseline – model without genotype-covariate interactions, Kernel – model with an environmental kernel matrix, RRR1 and RRR2 – reduced rank regression of rank one and two with observed covariates, FW1-US and FW2-US – random factorial regression with one and two synthetic covariates, RFR – random factorial regression with observed covariates. MVP for the baseline model was taken from the standard ASReml-R output.

| **Type** | **Model** | **MSPE** | | **MVP** | |
| --- | --- | --- | --- | --- | --- |
|  |  | **Mean** | **Median** | **Mean** | **Median** |
| With the main EC effect | Baseline | 1.22 | 0.744 | 1.10 | 1.10 |
|  | RRR1 | 1.22 | 0.816 | 1.17 | 1.17 |
|  | RRR2 | 1.22 | 0.810 | 1.17 | 1.17 |
|  | RFR | 1.22 | 0.804 | 1.16 | 1.16 |
|  | FW1-US | 1.22 | 0.863 | 1.11 | 1.12 |
|  | FW2-US | 1.23 | 0.851 | 1.08 | 1.08 |
| Without the main EC effect | Baseline | 1.22 | 0.824 | 1.12 | 1.12 |
|  | RRR1 | 1.22 | 0.851 | 1.13 | 1.13 |
|  | RRR2 | 1.22 | 0.851 | 1.12 | 1.13 |
|  | RFR | 1.22 | 0.839 | 1.12 | 1.12 |
|  | FW1-US | 1.22 | 0.846 | 1.12 | 1.12 |
|  | FW2-US | 1.22 | 0.846 | 1.12 | 1.13 |

**Table S9.** Variance of the predicted difference (VPD) and mean squared error of prediction difference (MSEPD) from the leave-one-year-and-location-out cross-validation (winter rice). Baseline – model without genotype-covariate interactions, Kernel – model with an environmental kernel matrix, RRR1 and RRR2 – reduced rank regression of rank one and two with observed covariates, FW1-US and FW2-US – random factorial regression with one and two synthetic covariates, RFR – random factorial regression with observed covariates.

| **Type** | **Model** | **MSEPD** | | **VPD** | |
| --- | --- | --- | --- | --- | --- |
|  |  | **Mean** | **Median** | **Mean** | **Median** |
| With the main EC effect | Baseline | 0.838 | 0.711 | 0.631 | 0.631 |
|  | RRR1 | 0.846 | 0.701 | 0.626 | 0.634 |
|  | RRR2 | 0.850 | 0.723 | 0.612 | 0.628 |
|  | RFR | 0.849 | 0.722 | 0.624 | 0.614 |
|  | FW1-US | 0.847 | 0.722 | 0.614 | 0.624 |
|  | FW2-US | 0.85 | 0.731 | 0.635 | 0.616 |
| Without the main EC effect | Baseline | 0.838 | 0.711 | 0.631 | 0.631 |
|  | RRR1 | 0.846 | 0.702 | 0.635 | 0.634 |
|  | RRR2 | 0.850 | 0.725 | 0.626 | 0.628 |
|  | RFR | 0.849 | 0.723 | 0.612 | 0.614 |
|  | FW1-US | 0.847 | 0.723 | 0.624 | 0.624 |
|  | FW2-US | 0.85 | 0.734 | 0.614 | 0.615 |


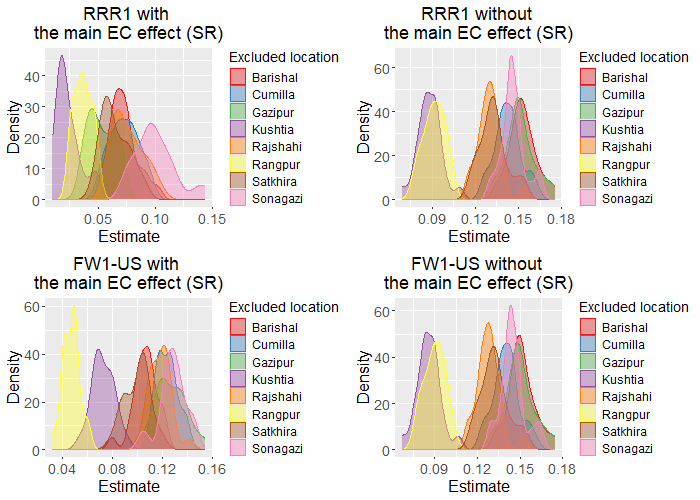


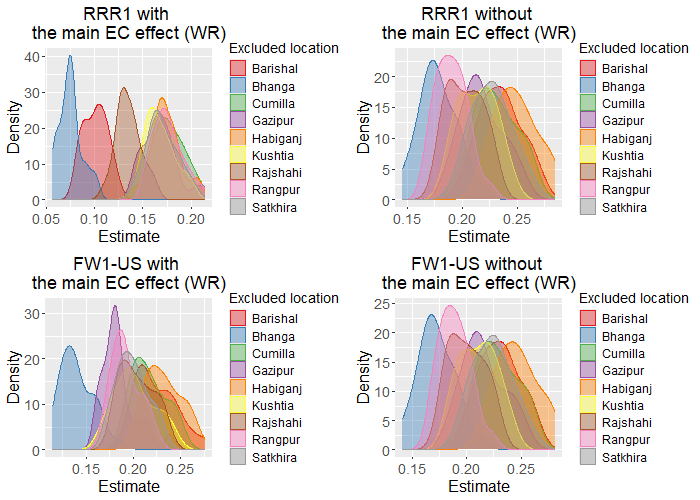


**Figure S1.** The distribution of the location (*L*) variance component estimate in leave-one-year-and-location-out (LYLO) cross-validation (CV) for the summer rice (SR) and the winter rice (WR) datasets. RRR1 and RRR2 – reduced rank regression of rank one and two with observed covariates, FW1-US and FW2-US – random factorial regression with one and two synthetic covariates.

**
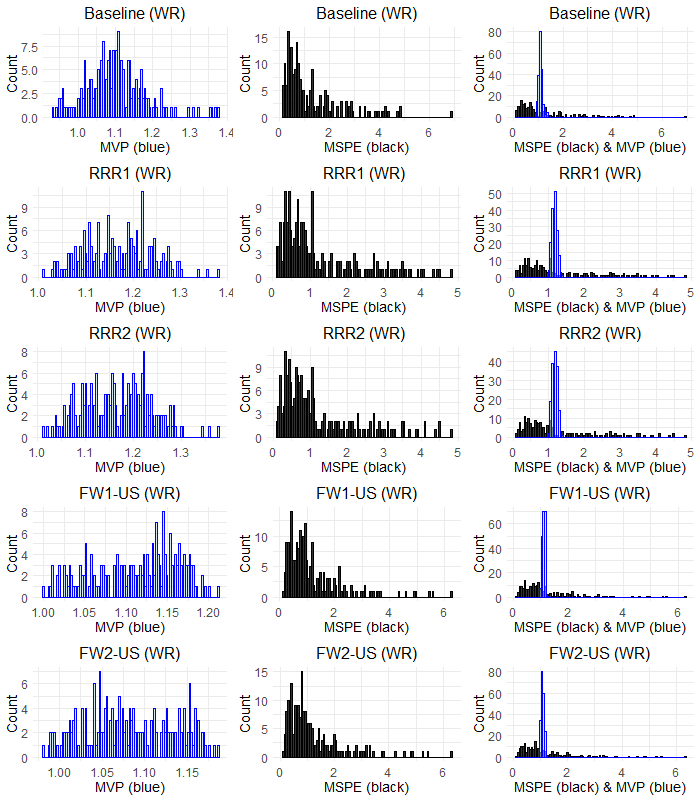
**

**Figure S2.** Distributions of model-based mean variance of prediction and cross-validation-based mean squared prediction error for all models with the main EC effect fitted to the winter rice (WR) data. Baseline – model without genotype-covariate interactions, RRR1 and RRR2 – reduced rank regression of rank one and two with observed covariates, FW1-US and FW2-US – random factorial regression with one and two synthetic covariates.

**
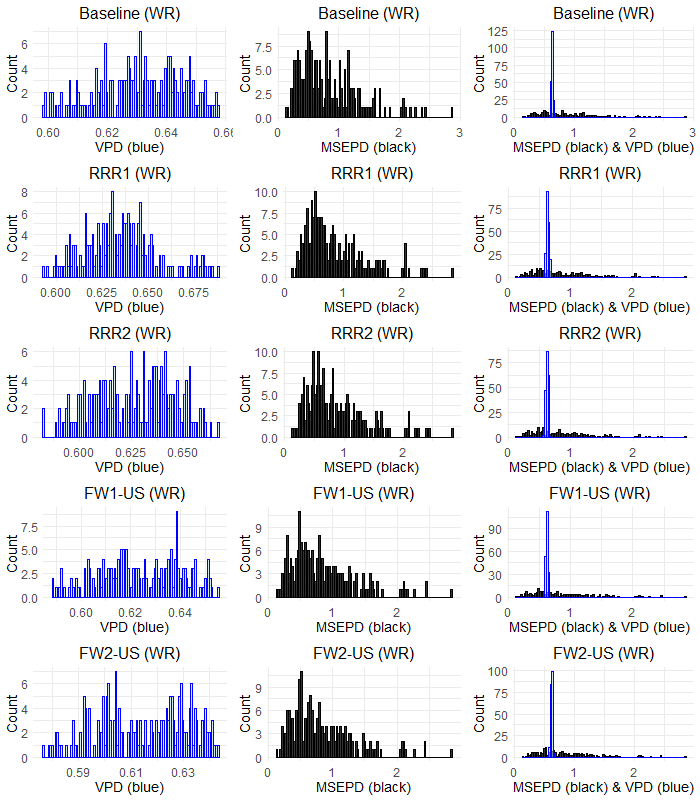
**

**Figure S3.** Distributions of model-based variance of predicted difference and cross-validation-based mean squared error of prediction difference for all models with the main EC effect fitted to the winter rice (WR) data. Baseline – model without genotype-covariate interactions, RRR1 and RRR2 – reduced rank regression of rank one and two with observed covariates, FW1-US and FW2-US – random factorial regression with one and two synthetic covariates.

**
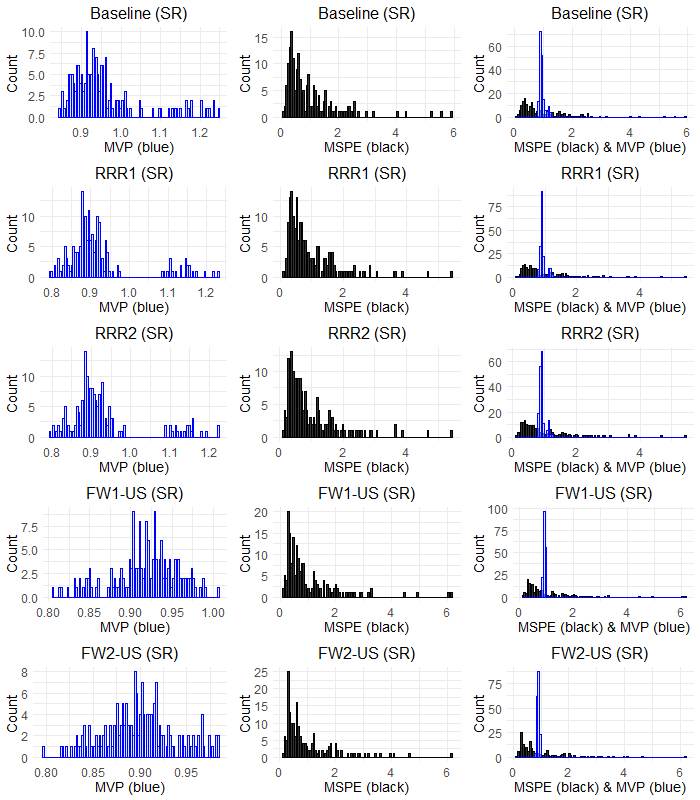
**

**Figure S4.** Distributions of model-based mean variance of prediction and cross-validation-based mean squared prediction error for all models with the main EC effect fitted to the summer rice (SR) data. Baseline – model without genotype-covariate interactions, RRR1 and RRR2 – reduced rank regression of rank one and two with observed covariates, FW1-US and FW2-US – random factorial regression with one and two synthetic covariates.

**
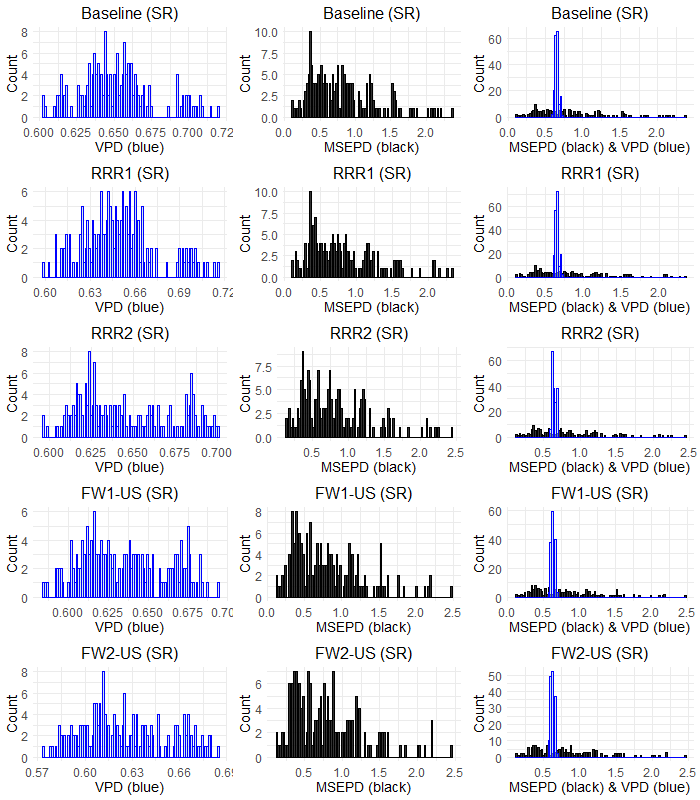
**

**Figure S5.** Distributions of model-based variance of predicted difference and cross-validation-based mean squared error of prediction difference for all models with the main EC effect fitted to the summer rice (SR) data. Baseline – model without genotype-covariate interactions, RRR1 and RRR2 – reduced rank regression of rank one and two with observed covariates, FW1-US and FW2-US – random factorial regression with one and two synthetic covariates.


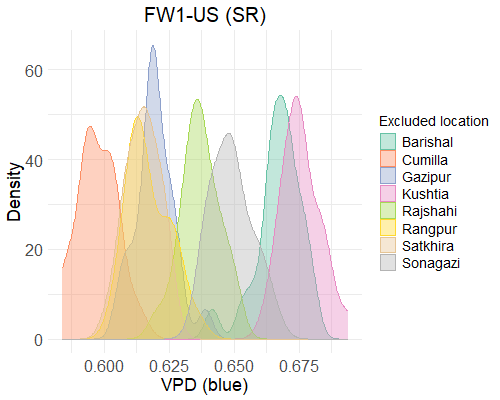


**Figure S6.** The bimodal distribution observed in Fig. S5 is due to exclusion of locations in the leave-one-year-and-location-out (LYLO) cross-validation approach. Different colors represent which location was excluded. VPD – variance of the predicted difference, FW1-US – random factorial regression with one synthetic covariate (with the main EC effect).
